# Predictive value of Heart Rate Variability measurements and the Brief Resilience Scale for workability and vitality

**DOI:** 10.3233/WOR-220366

**Published:** 2023-11-10

**Authors:** Marianne W.M.C. Six Dijkstra, Remko Soer, Hendrik J. Bieleman, Douglas P. Gross, Michiel F. Reneman

**Affiliations:** a Research Group Smart Health, Saxion University of Applied Sciences, Enschede, The Netherlands; b Department of Rehabilitation Medicine, University Medical Center Groningen, University of Groningen, Groningen, The Netherlands; c Department of Anaesthesiology and Pain Center, University Medical Center Groningen, University of Groningen, Groningen, The Netherlands; d Department of Physical Therapy, University of Alberta, Edmonton, Canada

**Keywords:** Prospective, primary prevention, occupational health, resilience

## Abstract

**BACKGROUND:**

Sustainable employability is increasingly important with current socio-economic challenges. Screening for resilience could contribute to early detection of either a risk, or a protector for sustainable employability, the latter being operationalized as workability and vitality.

**OBJECTIVE:**

To study the predictive value of Heart Rate Variability (HRV) measurements and the Brief Resilience Scale (BRS) for worker self-reported workability and vitality after 2–4 years.

**METHODS:**

Prospective observational cohort study with mean follow-up period of 38 months. 1,624 workers (18–65 years old) in moderate and large companies participated. Resilience was measured by HRV (one-minute paced deep breathing protocol) and the BRS at baseline. Workability Index (WAI), and the Vitality dimension of the Utrecht Work Engagement Scale-9 (UWES-9-vitality) were the outcome measures. Backward stepwise multiple regression analysis (*p* < 0.05) was performed to evaluate the predictive value of resilience for workability and vitality, adjusted for body mass index, age and gender.

**RESULTS:**

*N* = 428 workers met inclusion criteria after follow-up. The contribution of resilience, measured with the BRS, was modest but statistically significant for the prediction of vitality (R^2^ = 7.3%) and workability (R^2^ = 9.2%). HRV did not contribute to prediction of workability or vitality. Age was the only significant covariate in the WAI model.

**CONCLUSION:**

Self-reported resilience modestly predicted workability and vitality after 2–4 years. Self-reported resilience may provide early insight into the ability of workers to stay at work, although caution must be applied because explained variance was modest. HRV was not predictive.

## 1 Introduction

Keeping workers in the aging workforce is important because there is less recruitment of younger workers and also because work contributes to the well-being and financial independence of people in general [1]. The ability to adapt to adverse or changing situations might be a factor that explains why staying at work was not found to be related to calendar age in a previous observational study, despite cognitive and physical declines with age [2]. In an occupational context, circumstances change constantly as a natural result of technical and societal processes over time. Most workers seem to have appropriate compensation strategies that enable them to adapt and stay at work [2, 3]. Using these strategies to stay at work might be related to resilience [4], which is the ability to bounce back from adversity [4, 5]. Screening for resilience could contribute to early detection of impaired sustainable employability or as a protective factor for sustainable employability.

Measuring sustainable employability is difficult because it refers to a condition in the future, throughout and at the end of a worker’s career. In order to objectify sustainable employability for current workers, two elements are often measured: workability and vitality [6]. Workability is the degree to which workers assess their ability to perform their job [7], while vitality at work has been defined as mental resilience while working, the willingness to invest effort in one’s work, and persistence even in the face of difficulties [8].

Resilience can be measured using questionnaires or biometrics. Most resilience questionnaires focus on the reasons for reduced resilience [5]. The 6-item Brief Resilience Scale (BRS), however, was developed by Smith et al [9] to measure the range of resilience as a one factor structure, the ability to bounce back, without measuring reasons for reduced resilience [9]. The BRS is, therefore, also able to identify high resilience, the hypothesized protective factor for staying at work. A promising biometric measure for resilience is Heart Rate Variability (HRV), the natural variation in heart rate that reflects the Autonomic Nervous System (ANS) dynamics [10]. The ANS enables us to respond to stressful events. For instance, when a stressful event occurs, our heartbeat increases, adrenaline increases, we experience more alertness, thus enabling us to respond to the situation. A high HRV indicates a higher ability to respond effectively [11].

Interestingly, BRS and HRV have been found to be associated with resilience resources (e.g. BRS with coping, acceptance, humor [12]; HRV with better self-regulation, more social engagement [11]) as well as health related outcomes that are prevalent in the working population (e.g. HRV with coronary heart disease, diabetics and burnout [13–15]; BRS with neck and low back pain, somatic symptoms, depression [12, 16]). However, to our knowledge, the BRS and HRV have not been studied for their ability to predict measures of sustainable employability. This leads to our research question: what is the predictive value of Heart Rate Variability measurements and the Brief Resilience Scale for worker self-reported workability and vitality after two to four years?

## 2 Methods

### 2.1 Study design

A prospective observational follow-up study was carried out with baseline measurements between November 2015 and November 2016. Follow-up period was between 24 and 48 months.

### 2.2 Setting

Because the study aimed for applicable results within a real-life context, data were collected during routine Workers’ Health Assessments (WHA) conducted on regular clients of an Occupational Healthcare Supplier (OHS) servicing 18 moderate and large companies in a variety of sectors (education, transport, automotive industry, housing association, water board, food processing, waste processing, metal industry, retail, finance) in the Netherlands. The WHAs were performed by the independent OHS to promote sustained employability, and were used to identify specific work-related health risks in order to prevent injuries or disease through intervention [17]. All workers from participating companies were invited. Participation was voluntary, and companies were responsible for the invitation process. Legislation in the Netherlands requires companies to offer employees a WHA at least every four years. Therefore, our maximum follow-up period was four years. The minimum follow-up period was two years, which was sufficient to allow health changes to occur.

### 2.3 Ethical standards

The Ethics Board at the University Medical Center Groningen in the Netherlands confirmed (August 20, 2015) that formal approval of the study was not necessary because all workers were only subjected to routine care. The study was performed in accordance with the Declaration of Helsinki of 1964 and its later amendments. Participants signed informed consent forms stating that their anonymized data could be used for scientific purposes to improve the quality of the WHA service. This paper adhered to the EQUATOR network STROBE guidelines for observational cohort studies.

### 2.4 Participants

All workers (from participating companies described in the Setting section) between 18 and 65 years old were included. Workers absent from work at the time of the measurements did not participate. Pregnant women were excluded. Workers with pacemakers or beta-blockers were excluded because of the influence on HRV.

### 2.5 Measurements

#### 2.5.1 Demographic variables, health and work characteristics at baseline

Personal (age, gender, level of education) and work characteristics (working hours per week, number of years employed, working in shifts, type of contract) were collected with an online questionnaire by a secured web-based application. After completion, health characteristics (systolic and diastolic blood pressure, body mass index (BMI), cholesterol, glucose), were measured and registered by the OHS. Workability and vitality were measured at baseline as health characteristics and at follow-up as dependent variables.

#### 2.5.2 Dependent variables at follow-up (24–48 months)

Workability and Vitality were measured with the following questionnaires.

1. Workability was measured using the Work Ability Index (WAI) [7, 18, 19]. With this questionnaire, workers assess the degree to which they consider themselves physically and mentally able to perform their work. In addition, norms and values, competencies and health are part of the WAI. The WAI consists of 7 items: 1) job characteristics and workability related to work demands (2–10 points); 2) current workability compared to lifetime best workability (0–10 points); 3) current conditions/illnesses (1–7 points); 4) estimated limitation of working capacity due to condition/illnesses (1–6 points); 5) sick-leave during the last 12 months (1–5 points); 6) personal estimated workability in future 2 years (1–7 points); and 7) vitality (1–4 points). The scale ranges between 7–49, with higher scores indicating better workability. The WAI has been shown to be valid and reliable [18] and highly predictive for prolonged sickness absence and disability pension [18, 19] in workers with poor or moderate work ability.
2. Vitality was measured using the subscale ‘vitality’ (also referred to as ‘vigor’) of the Utrecht Work Engagement Scale-9 (UWES-9), using three questions with a 0-to-6 point scale per question: 1) ‘At my work, I feel bursting with energy’, 2) ‘At my job, I feel strong and vigorous’, 3) ‘When I get up in the morning, I feel like going to work’. Total score is divided by three and ranges from 0-to-6, with a higher score indicating more vitality [8]. The UWES-9-vitality subscale has good internal consistency: median value of Cronbach’s *α* over 25 studies from the Netherlands and Belgium is.83 [8].

#### 2.5.3 Independent variables at baseline

Resilience was measured with biometry and a questionnaire:

1. Heart Rate Variability (HRV) was used as a proxy-marker for resilience and measured with the ear lobe pulse sensor (photo pletysmography (PPG) technology) of the emWave® Pro Plus (Heartmath). PPG technology is a reliable and valid method of capturing and quantifying HRV during a deep breathing test [20]. Participants were instructed to remain seated, relaxed and to refrain from making any significant or rapid body movements. They were instructed to breathe according to the one-minute, 6-breath protocol that was paced at a rhythm of six breaths per minute (0.1 Hz) while breathing as deeply as they comfortably could. This breathing method was designed to provide a physiological challenge to assess a person’s maximum HRV range [21]. Breathing rhythm is an important confounder in ultrashort HRV measurement and was therefore standardized by the use of a visual breath pacer set at six breaths per minute. The entire minute must be artefact-free. A second test was performed when the quality of the attempt was questioned [22]. The Mean Heart Rate Range (MHRR) was used as outcome because it has been suggested as an indicator of autonomic nervous system health [21], with the practical advantage that it can be explained easily to workers. This time domain variable of HRV represents the magnitude of the amplitudes in acceleration and deceleration of the heart. The EmWave Pro Plus® software calculated MHRR according to the formula $(\Sigma_{i=1}^{6}({\mathrm{HF}}_{\max(i)}-{\mathrm{HF}}_{\min(i)}))/6$ ; *i* = breathing cycle.
2. The questionnaire to measure resilience was the Dutch language version of the Brief Resilience Scale (BRS-DLV; Appendix A) [23]. The BRS-DLV contains six items scored on a 5-point Likert scale from 1 = strongly disagree to 5 = strongly agree. The total score is the mean score of all answers and thus ranged from 1 to 5. Higher scores indicate better resilience. A factor analysis showed that the BRS-DLV is unidimensional with a variance of 49% –67% explained by this factor [23]. Cronbach’s *α* was between 0.80 and 0.91 and test-retest reliability (ICC) was 0.69 for 1 month and 0.62 for 3 months [9].

#### 2.5.4 Covariates

MHRR measured using the one-minute paced-breathing protocol decreased with older age and/or higher BMI [22]. HRV was also found to correlate with gender: in a 24-hour HRV measurement, HRV was lower in female than male subjects younger than 30 years; gender differences decreased between 30 and 50 years old and disappeared after age 50 [24]. No covariates of the BRS (Dutch or English version) are known. Therefore, age, BMI and gender were analyzed covariates in the prediction model.

### 2.6 Data handling

Questionnaire data were entered in the OHS company software by the worker and scores were processed automatically according to each questionnaires guidelines, except for the BRS-DLV. Patient characteristic and HRV biometry results were entered in the same software manually by the OHS. All data were then compiled into an Excel file and de-identified by the OHS. The data were then sent to the researchers.

The researchers processed the data of the BRS-DLV, which is constructed with three reverse phrased questions, for example “I tend to take a long time to get over set-backs in my life” to enable detection of an inconsistent pattern. These answers were reversed in SPSS during data-processing. In line with the validation study of the BRS-DLV [23], the researchers manually removed inconsistent cases that indicated respondents either did not understand or read the responses. Only cases with complete data for WAI-T1, UWES-9-Vitality-T1, HRV-T0, BRS-T0, age, gender and correct follow-up period were used for statistical analysis (T0 = baseline, T1 = follow-up). Missing data are presented in the result section.

MHRR data were categorized according to data handling description in a previous study [22]: “..the data were divided into subgroups because literature indicates low HRV is related to health problems [11, 13, 15]. In absence of validated meaningful cut-off values, statistical criteria were used to determine cut-off values. Because only low scores are known to have a clinical value [11, 13, 15], cut-off values are provided on the left side of the normal distribution of MHRR of a healthy reference group, corrected for age (proprietary; Emwave Pro Plus® software): very low MHRR when MHRR > 2 SD below mean, low HRV when MHRR is 1-2 SD below mean, (above) average HRV when the deviation is higher than 1 SD below mean.”

### 2.7 Statistical analysis

Descriptive statistics were calculated for all variables. A large number of workers (*n* = 1,196) were excluded due to missing data or a follow-up period < two years. To test for selection bias, differences between the included and excluded group were compared for demographic, personal and health variables. A *t*-test with Bias-corrected and accelerated (BCa) bootstrap procedure (1,000 samples) was used for continuous data for continuous data (*P* < .05). A Kruskal-Wallis H test was used for ordinal and nominal data. Difference between the HRV groups was tested with a Kruskal-Wallis H test, followed by a pairwise comparison.

To assess the joint predictive ability of HRV, BRS-DLV and the covariates (BMI, gender and age) on workability and vitality, yet eliminating the least important variables, a backward stepwise multiple regression analysis (*p* < .05) was used. The 95% Confidence Interval around B is calculated with a BCa bootstrap procedure (1,000 samples) to overcome bias from heteroscedasticity and normality of residuals. With the backward procedure, the least significant variable was excluded from the model step by step until all remaining variables contributed significantly (*p* < .05) and collinearity was acceptable (Variance Inflation Factor ≈ 1). The backward stepwise multiple regression procedure is preferred to a forward procedure, because all variables are taken into account simultaneously and multi-collinearity is accounted for. Sensitivity analyses for HRV was performed by entering raw MHRR data in the regression analysis. IBM-SPSS (version 26) software was used for statistical analysis.

## 3 Results

### 3.1 Participants

At baseline, 1,624 workers employed at 18 companies participated; 630 were measured before the follow-up period of 24 months and were therefore excluded. Another 566 cases were excluded because they missed (a combination of) variables (143 workers missed the UBES-9-vitality-T1; 75 the WAI-T1; 191 the MHRR-T0; 140 the BRS-score-T0). Six workers were excluded because the BRS data were inconsistent. This led to 428 included workers employed at four companies for the final modeling. WAI and UBES baseline score of the included and excluded group were compared to rule out selection bias; the groups did not differ significantly (*p* < .01). The majority (*n* = 402) worked at a holding company in the automotive industry. The others (*n* = 26) were employed at a housing association, meat processing plant or water board company.

### 3.2 Personal, work and health characteristics at baseline

Personal and work characteristics at baseline are presented in Table 1. Mean age was 41 years (SD 9.5); the majority of participants were male. Most workers had a fixed contract, did not work in shifts and level of education was intermediate or high. Median working years was 7.5 years with a right-skewed distribution, and median working hours a week was 37.6 hours with a left skewed distribution. The included group was significantly higher educated, worked more in mentally demanding work and less in shifts than the excluded group (*p* < 0.05). There were more females in the included group. Other differences were minor and not relevant or significant. Results of both included and excluded participants are presented in Appendix B.

**Table 1:** Personal and work characteristics at baseline of the included workers

Personal and work characteristics at baseline		Included workers (*n* = 428)
Age in years, mean (*SD*)	41 (9.5)
Gender, %	Male	77.3 %
	Female	22.7 %
Education level, %	Very low	0.5 %
	Low	7.2 %
	Intermediate	39.7 %
	High	52.1 %
	Missing	0.4 %
Type of work, %	Physically demanding	7.9 %
	Mentally demanding	75.9 %
	Physically and mentally demanding	13.6 %
	Missing	2.6 %
Irregular shifts, %	Yes	17.8 %
	No	81.8 %
	Missing	0.4 %
Contract, %	Fixed	89.7 %
	Temporary/Flexible hours	9.8 %
	Missing	0.4 %
Working hours/week, median (min–max)		37.6 (1–55)
	Missing, *n*	2
Years employed, median (min–max)		7.5 (0–45)
	Missing, *n*	2

Level of education: very low = no or elementary education; low = lower vocational education; intermediate = intermediate vocational education and high school; high = bachelor or higher education.

Health characteristics at baseline are shown in Table 2. Median BRS score was 3.6 (range 2.0–5.0) and median MHRR was 22.0 beats/minute (range 2.8 – 70.0 beats/minute). The Kruskall-Wallis H test showed that groups differ significantly (*p* < .001). However, the pairwise comparison showed that the very low and low MHRR group did not differ (*p* = .45). The (above) average group was large, as a result of the categorization procedure. No relevant differences in health characteristics at baseline were found between the included and excluded workers (Appendix C). Univariate partial correlation between BRS and MHRR, controlled for age, was.04 (BCa 95% interval: –.05 to.13).

**Table 2:** Participant health characteristics at baseline

Baseline health characteristics	Included workers (*n* = 428)
Median (min–max), *n*
Workability: Work Ability Index score (range 7–49)	43 (23–49), 416
Vitality: UWES-9-vitality items, (range 0–6))	4.3 (1.3–6.0), 428
Resilience: Brief Resilience Scale score (range 1–5)	3.6 (2.0–5.0), 428
Resilience: Heart Rate Variability – Mean Heart Rate Range (beats/minute)	22.0 (2.8–70.0), 428
Very low HRV – Mean Heart Rate Range (beats/minute)	5.7 (2.8–8.1), 20
Low Mean HRV – Heart Rate Range (beats/minute)	8.6 (7.0–11.9), 25
(Above) average HRV – Heart Rate Range (beats/minute)	23.7 (7.6–70), 383
Blood Pressure Diastolic (mmHg)	80.0 (55–121), 428
Blood Pressure Systolic (mmHg)	133.5 (96–186), 428
Blood Cholesterol (mmol/l)	5.1 (2.6–9.3), 422
Blood Glucose (mmol/l)	5.1 (1.3–11.9), 422
Body Mass Index (kg/m^2^)	25.9 (17.1–38.7), 427
Waist circumference (cm)	92.1 (69–125), 420

### 3.3 Dependent variables at follow-up

Median duration between T0 and T1 was 38.4 months (minimum 24–maximum 46 months). With a median WAI-score of 42.7, workability at follow-up was good; the median UWES-9-vitality score was 4.2, indicating a moderate vitality (Table 3).

**Table 3:** Dependent variables of the included workers at follow-up (*n* = 428) after 2–4 years

Dependent variables at follow-up (*n* = 428)
	Median (min–max)
Workability Index Score (range 7–49)	42.7 (24–49)
Utrecht Work Engagement Scale-9-vitality score (range 0–6)	4.2 (1.3–6.0)

### 3.4 Regression analysis

Table 4 shows the final models for WAI-score and for UWES-9-vitality score. MHRR was excluded in the second step, after gender, for the Workability model and in the first step for the UWES-9-vitality model. All steps of the linear backward regression procedure can be found in Appendix D. The variance inflation factor (VIF) values were below 1.1 in all steps and 1.0 in the final models, indicating there was no collinearity between variables. The sensitivity analyses with raw MHRR data (results not shown in detail) revealed a similar picture. MHRR in the very low and low categories did not differ significantly; therefore we additionally tested the effect of merging these groups, which did not change the outcome (results not shown in detail). MHRR did not contribute significantly to prediction of Workability or UWES-9-vitality. In the final models, the contribution of resilience, measured using the BRS-DLV, was modest but significant for the prediction of WAI-score (R^2^ = 9.2%) and UWES-9-vitalty score (R^2^ = 7.3%). Age was included as a significant co-variate only in the model for WAI. All other variables were excluded for the final models.

**Table 4:** Results of the linear multiple regression analysis with backward procedure for Work ability index score and UWES-9-vitality score at follow-up

Dependent variable	Model	Unstandardized B	Standardized B	BCa 95.0% for B						
				Lower Bound	Upper Bound	Std. Error	Sig.	R^2^	Adjusted R^2^	VIF
Workability Index	(WAI)						<.001	9.2%	8.8%
	Constant	39.275		36.098	42.246	1.580	.001
	BRS-DLV score	1.826	.249	1.151	2.504	.338	.001			1.000
	Age	–.077	–.172	–.120	–.032	.022	.001			1.000
Vitality							<.001	7.3%	7.0%
(UWES-9-vitality)
	Constant	2.523		1.882	3.154	.326	.001
	BRS-DLV score	.461	.296	.293	.634	.086	.001			1.000

BRS-DLV=Brief Resilience Scale-Dutch Language Version. UWES = Utrecht Work Engagement Scale; the construct vitality of this scale was used.

## 4 Discussion

This cohort study assessed the ability of measures of resilience to predict future sustainable employability. Results show that resilience, measured using the BRS-DLV, contributes modestly but statistically significant to prediction of workability and vitality. In fact, it was the only variable that remained in both final models. We did not observe a significant contribution of HRV in prediction of workability or vitality. The only covariate that contributed to one of the final models was age, which, consistent with previous research [25], was negatively associated with future workability.

We did not observe predictive value of MHRR for workability or vitality. A possible explanation might be the difference in measure characteristics. It has been shown that self-report measures and biometric measures for the same construct correlate only moderate [26]. This might be more evident in a relatively healthy population with little variation in outcomes and low prevalence of (very) low HRV (4.6%). Prevalence is low because we used non-clinically set, reasoned cut-off scores. The protocol with MHRR as an outcome is used in other settings as part of Ewing’s test-battery to screen for dysfunctional autonomic nervous system in persons with diabetes-type II [27]. For this test-battery the cut-off values vary: 15–20 beats/minute (age < 50 years) and 5 beats/minute (age > 50 years) [27]. The cut-off values of MHRR in our study are lower as compared to Ewing’s battery. We are not aware of studies or guidelines with cut-off scores for better health outcomes with higher HRV values in the (above) average range, and therefore the (above) average group is large. The question arises whether the one-minute paced-breathing protocol with the non-clinically set cut-off values is sensitive enough for our relatively healthy workers [15]. Other studies that reported associations between different health conditions and low HRV, used longer HRV-protocols and other measures (e.g., Root Mean Square of Successive Differences (RMSSD)) [13–15]. These protocols are not practical as part of a WHA, and correlation between MHRR and other measures of HRV is good (*r* = 0.66 with RMSSD) [20, 22], which supports our choice of the short protocol and MHRR. However, using the paced-breathing protocol is new for prediction of workability and vitality in healthy workers. It would be interesting to study clinically relevant cut-off values for HVR for the protocol in future studies.

Our other finding, the predictive value of BRS-DLV for workability and vitality, supports previous work hypothesizing that staying at work might be related to the ability to adapt to the constantly changing circumstances of working life [2, 4, 28]. There has been much research on how to support the workforce to stay at work, but most studies address sustainable employability by determining or preventing risks of turnover, early retirement or disability [29, 30], which are important preconditions for staying at work. However these studies do not actually focus on *preserving* health and enabling workers to stay at work through working life. Our study contributes to the growing body of knowledge for the enabling factors contributing to stay at work, which are not necessarily the opposite of risks for leaving work [31, 32].

An interesting concept to discuss regarding resilience at work is coping, which previously has been found associated with workability [18, 33]. Although the concepts are often used interchangeably [34], coping can be defined as a wide set of (positive or negative) skills and cognitive and behavioural efforts in response to stress, while resilience can be seen as a result of a favorable coping style [4, 34]. Kadijk et al. concluded that unfavorable coping styles for employees with combined mental and physical health problems are harmful for work ability [33]. In line with a previous study [18], they suggested that interventions to improve workability should be focused on favorable coping styles, such as resilience, to deal with physical and mental requirements rather than addressing working conditions or health seperately.

In a recent meta-analysis, resilience interventions based on a combination of cognitive behavioural therapy and mindfulness techniques appeared to have a positive impact on individual resilience [35]. Another review showed that the effect of resilience training in a working context seemes to diminish over time, but increases in populations at greater risk of experiencing stress and lacking core protective factors [36].

### 4.1 Strenghts and limitations

The strengths of our study are that we followed *regular occupational health* services of a *large*, *heterogenuous* working population (*n* = 428) and *a variety of companies*, increasing the likelihood of generalizable results. Additionally, we studied two measures of resilience.

Observational research also has weaknesses that can affect results. One limitation of this design was a potential for selection bias. Nine companies chose to follow-up within 24 months and were therefore excluded from the study. At baseline, WAI and UBES-9-vitality scores between companies excluded for this reason, did not differ significantly from the included companies. Therefore we concluded that unfavourable score on WAI or UBES-9-vitality at baseline did not influence the follow-up period chosen by the companies. Another potential selection bias was set by the option to use a different set of questionnaires for corporate motives, which occurred in four of the remaining companies. To examine whether there was selection bias by not administering these questionnaires at follow-up to workers with an undesirable WAI or UBES-9-score, we tested for a difference at baseline for workers with and without WAI or UBES-9-vitality variables at follow-up. There appeared to be no statistically significant differences, and this loss to follow-up was unlikely to be related to baseline WAI or UBES-9-vitality score. It is likely that sampling bias has influenced results because workers on sickleave were not included, causing a healthy-worker effect. Previous studies have demonstrated that poor health [37], sickness absence and presenteeism [38] were associated with unsustainable employability. The excluded workers in our study were lower educated and had more physically demanding work, which are known risk factors for reduced sustainable employability [37, 39]. Missing workers at high risk of unsustainable employability, might have decreased the explained variance we found due to the ceiling effect of the WAI and UBES-9-vitality-score. Finding a modest, but significant explained variance in a selected group of healthy workers, can be seen as a promising finding for early detection of the presence of a protective factor for sustainable employability. Methodological bias has likely influenced the positive relation we observed between vitality and the BRS-DLV.

As mentoined before, mental resilience is part of the definition of vitality and it is highly likely that it associates with resilience measured with the BRS-DLV. A conceptual difference between the two is that UWES-9-vitality explicitely focusses on vitality *at work* and the three questions do not seem to reflect the definition of resilience, while the BRS-DLV is developped to measure resilience in general [9]. It is possible that workers are vital for work in early stages of health conditions. In the recent dynamic view of health as ‘the ability to adapt and to self-manage, in the face of social, physical and emotional challenges’ [40], the human capacity for resilience and coping with new situations is highlighted [40]. Therefore we decided to screen for general resilience as measured with the BRS-DLV for early prediction of workability and vitality. A final confounder could be that the influence of lifestyle activities to HRV results (e.g., poor sleep quality, alcohol consumption or coffee intake [27]) cannot be ruled out, even though participants were asked to avoid them prior to testing.

The field of studying factors contributing to staying at work (which is not the opposite of risks for leaving work) is relatively young, but very important for wellbeing of the aging workforce [2, 31]. With more knowledge about stay-at-work factors, early interventions could be developed or applied to support workers. This is parallel to managing risk factors for leaving work, which is already performed in most companies. Future studies with longer follow-up periods in large cohorts are required to gain the necessary insight into factors supporting workers. Studies with longer follow-up periods might also be of additional value to detect the sensitive periods when interventions are most successful.

## 5 Conclusion

This study adds to the growing body of research about workers’ protective factors for sustainable employability. The BRS-DLV was modestly predictive for both workability and vitality after two-to-four years follow-up. HRV measured was not predictive for workability or vitality. The present study raises the possibility that screening for self-reported resilience may contribute to early insight into the ability of workers to stay at work when resilience is high, although caution must be applied because contributing variance was modest. We suggest that screening workers for resilience with the BRS may be helpfull to detect early signs of decreased favourable coping styles.

## Ethical disclosure

*Ethical approval:* The Ethics Board at the University Medical Center Groningen in the Netherlands confirmed (August 20, 2015) that formal approval of the study was not necessary because all workers were only subjected to routine care. The study was performed in accordance with the Declaration of Helsinki of 1964 and its later amendments.

*Ethical authorship:* All authors declare to meet all ethical criteria of authorship. The manuscript has been read and approved for representing honest work by all authors. All authors contributed to the study conception and design. The first draft of the manuscript was written by MSD and all authors critically commented on previous versions of the manuscript.

*Informed consent:* Participants signed informed consent forms stating that their anonymized data could be used for scientific purposes to improve the quality of the WHA service.

*Registry of study:* The study was registered at the Netherlands Trial Register – Trial NL 7337 (NTR7553).

## Conflict of interest

The authors declare they have no relevant financial or non-financial conflict of interests related to the content of this article. The funding source had no involvement in the study design, data collection, analysis or interpretation, in the writing of the report, or the decision to submit the article for publication.

## Supplementary Material

Supplementary Material

## Supplementary materials

The appendices are available from https://dx.doi.org/10.3233/WOR-220366.
