# Supplementary material for: Predictive value of Heart Rate Variability measurements and the Brief Resilience Scale for workability and vitality
Source: Work. 2023 Nov 10;76(3):1007–17. doi: 10.3233/WOR-220366 (PMC10657665; doi:10.3233/WOR-220366)
Supplement: Supplementary Material [file wor-76-wor220366-s001.docx]

**Appendix A. Brief Resilience Scale**

**Brief Resilience Scale (Smith et al, 2018)**

Please indicate the extent to which you agree with each of the following statements by using the following

scale: 1 = strongly disagree, 2 = disagree, 3 = neutral, 4=agree, 5=strongly agree

|  |  |  |  |  |  |
| --- | --- | --- | --- | --- | --- |
| 1. I tend to bounce back quickly after hard times | 1 | 2 | 3 | 4 | 5 |
| 2. I have a hard time making it through stressful events | 1 | 2 | 3 | 4 | 5 |
| 3. It does not take me long to recover from a stressful event | 1 | 2 | 3 | 4 | 5 |
| 4. It is hard for me to snap back when something bad happens | 1 | 2 | 3 | 4 | 5 |
| 5. I usually come through difficult times with little trouble | 1 | 2 | 3 | 4 | 5 |
| 6. I tend to take a long time to get over set-backs in my life (R) | 1 | 2 | 3 | 4 | 5 |

**Brief Resilience Scale - Dutch Language Version:**

**Korte Veerkracht Vragenlijst (Nederlandse vertaling, Soer et al, 2018)**

Graag aangeven in hoeverre u het eens bent met de volgende stellingen, maak daarbij gebruik van de volgende schaal:

1 = sterk mee oneens, 2= mee oneens, 3 = neutraal, 4 = mee eens, 5 = zeer mee eens

| 1. Na een moeilijke periode veer ik meestal gemakkelijk weer terug | 1 | 2 | 3 | 4 | 5 |
| --- | --- | --- | --- | --- | --- |
| 2. Ik vind het moeilijk om me door stressvolle gebeurtenissen heen te slaan (R) | 1 | 2 | 3 | 4 | 5 |
| 3. Het kost me niet veel tijd om te herstellen van een stressvolle gebeurtenis | 1 | 2 | 3 | 4 | 5 |
| 4. Ik vind het moeilijk om het snel van me af te schudden als er iets ergs is gebeurd (R) | 1 | 2 | 3 | 4 | 5 |
| 5. Ik sla me meestal redelijk probleemloos door moeilijke periodes heen | 1 | 2 | 3 | 4 | 5 |
| 6. Het kost me meestal veel tijd om over tegenslagen in mijn leven heen te komen. (R) | 1 | 2 | 3 | 4 | 5 |

**Appendix B. Personal and work characteristics of included and excluded workers.**

| **Personal and work characteristics at baseline** |  | **Included Workers (n=428)** | **Excluded workers (n=1196)** |
| --- | --- | --- | --- |
| Age in years, mean (sd)* | | 41 (9.5) | 45 (10.2) |
|  | Missing, *n* | - | 30 |
| Gender* | Male | 77.3 % | 85.8 % |
|  | Female | 22.7 % | 14.2 % |
| Education level* | Very low | 0.5 % | 2.5 % |
|  | Low | 7.2 % | 16.6 % |
|  | Intermediate | 39.7 % | 40.2 % |
|  | High | 52.1 % | 36.9 % |
|  | Missing | 0.4 % | 3.8 % |
| Type of work* | Physically demanding | 7.9 % | 11.7 % |
|  | Mentally demanding | 75.9 % | 56.9 % |
|  | Physically and mentally demanding | 13.6 % | 19.8 % |
|  | Missing | 2.6 % | 11.6 % |
| Irregular shifts* | Yes | 17.8 % | 28.7 % |
|  | No | 81.8 % | 67.6 % |
|  | Missing | 0.4 % | 3.8 % |
| Contract* | Fixed | 89.7 % | 91.2 % |
|  | Temporary/Flexible hours | 9.8 % | 5.3 % |
|  | Missing | 0.4 % | 3.5 % |
| Working hours/ week, median (min-max) | | 37.6 (1-55) | 37.8 (0 - 48) |
|  | Missing, *n* | 2 | 45 |
| Years employed, median (min-max)* | | 7.5 (0-45) | 10.8 (0 - 50) |
|  | Missing, *n* | 2 | 45 |

*Level of education: very low = no or elementary education; low= lower vocational education; intermediate = intermediate vocational education and secondary higher level education; high = bachelor or higher education.*

**Appendix C. Baseline health characteristics of included and excluded workers.**

| **Baseline health characteristics** | **Included workers (n=428)** |  | **Excluded workers (n=1117)** | |
| --- | --- | --- | --- | --- |
|  | Median (min-max) | *(n)* | Median (min-max) | *(n)* |
| Work Ability Index score (range 7-49)* | 43 (23-49) | *(416)* | 42 (21-49) | (1,052) |
| Vitality (UBES 3 items, range 0-6)) | 4.3 (1.3-6.0) | *(428)* | 4.2 (1.0-6.0) | (887) |
| Brief Resilience Scale score (range 1-5) | 3.6 (2.0-5.0) | *(428)* | 3.6 (1.7-5.0) | (818) |
| Heart Rate Variability - Mean Heart Rate Range (beats/minute) | 22.0 (2.8 – 70.0) | *(428)* | 20.8 (2.1 – 84.0) | (727) |
| Blood Pressure Diastolic (mmHg)* | 80.0 (55-121) | *(428)* | 83.1 (48-127) | (1091) |
| Blood Pressure Systolic (mmHg)* | 133.5 (96-186) | *(428)* | 136.9 (89-207) | (1091) |
| Blood Cholesterol (mmol/l)* | 5.1 (2.6-9.3) | *(422)* | 4.9 (2.6-8.7) | (1065) |
| Blood Glucose (mmol/l) | 5.1 (1.3-11.9) | *(422)* | 5.1 (2.0-17.3) | (1064) |
| Body Mass Index (kg/m^2^) | 25.9 (17.1-38.7) | *(427)* | 26.2 (18.0-46.4) | (1101) |
| Waist circumference (cm)* | 92.1 (69-125) | *(420)* | 94.8 (67-146) | (1061) |

**groups differ significantly with p ≤ 0.05; an independent T-test with bootstrap analysis was performed. The number of cases for each of the variables were not complete and are note*

**Appendix D. All steps of the linear multiple regression analysis with backward procedure.**

All steps of the multiple linear regression analysis with backward procedure.

Dependent variable: Work Ability Index score.

| Step | Model | Unstandardized B | Standardized B | BCa 95.0% for B | |  |  |  |  |  |
| --- | --- | --- | --- | --- | --- | --- | --- | --- | --- | --- |
|  |  |  |  | Lower Bound | Upper Bound | Std. Error | Sig. | R^2^ | Adjusted R^2^ | VIF |
| 1 |  |  |  |  |  |  |  | 10.6% | 9.5% |  |
|  | Constant | 40.156 |  | 35.711 | 44.720 | 2.216 | 0.001 |  |  |  |
|  | BRS-DLV score | 1.718 | 0.234 | 1.031 | 2.439 | 0.339 | 0.001 |  |  | 1.022 |
|  | Gender | 0.263 | 0.026 | 0.593 | 1.368 | 0.485 | 0.593 |  |  | 1.020 |
|  | Age | -0.062 | -0.138 | 0.014 | -0.021 | 0.024 | 0.014 |  |  | 1.081 |
|  | BMI | -0.104 | -0.082 | 0.070 | 0.004 | 0.058 | 0.070 |  |  | 1.064 |
|  | MHRR | .0741 | 0.082 | 0.151 | 1.754 | 0.529 | 0.151 |  |  | 1.056 |
| 2 |  |  |  |  |  |  |  | 10.5% | 9.7% |  |
|  | Constant | 40.215 |  | 35.676 | 45.093 | 2.274 | 0.001 |  |  |  |
|  | BRS-DLV score | 1.737 | 0.236 | 1.076 | 2.418 | 0.339 | 0.001 |  |  | 1.013 |
|  | Age | -0.063 | -0.139 | -0.112 | -0.019 | 0.024 | 0.012 |  |  | 1.080 |
|  | BMI | -0.100 | -0.079 | -0.211 | 0.013 | 0.055 | 0.063 |  |  | 1.025 |
|  | MHRR | 0.738 | 0.082 | -0.131 | 1.725 | 0.525 | 0.153 |  |  | 1.056 |
| 3 |  |  |  |  |  |  |  | 9.9% | 9.2% |  |
|  | Constant | 41.910 |  | 37.600 | 45.8508 | 2.223 | 0.001 |  |  |  |
|  | BRS-DLV score | 1.776 | 0.242 | 1.059 | 2.468 | 0.361 | 0.001 |  |  | 1.008 |
|  | Age | -0.070 | -0.155 | -0.115 | -0.026 | 0.022 | 0.001 |  |  | 1.039 |
|  | BMI | -0.107 | -0.084 | -0.237 | 0.019 | 0.056 | 0.060 |  |  | 1.048 |
| 4 |  |  |  |  |  |  | <.001 | 9.2% | 8.8% |  |
|  | Constant | 39.275 |  | 36.098 | 42.246 | 1.580 | .001 |  |  |  |
|  | BRS-DLV score | 1.826 | .249 | 1.151 | 2.504 | .338 | .001 |  |  | 1.000 |
|  | Age | -.077 | -.172 | -.120 | -.032 | .022 | .001 |  |  | 1.000 |

*BRS-DLV = Brief Resilience Scale-Dutch Language Version. BMI = Body Mass Index. MHRR = Mean Heart Rate Range, the measure for Heart Rate Variability.*

All steps of the multiple linear regression analysis with backward procedure.

Dependent variable: UWES-9 Vitality score

| Step | Model | Unstandardized B | Standardized B | BCa 95.0% for B | |  |  |  |  |  |
| --- | --- | --- | --- | --- | --- | --- | --- | --- | --- | --- |
|  |  |  |  | Lower Bound | Upper Bound | Std. Error | Sig. | R^2^ | Adjusted R^2^ | VIF |
| 1 |  |  |  |  |  |  |  | 7.5% | 6.4% |  |
|  | Constant | 2.325 |  | 1.174 | 3.520 | 0.571 | 0.001 |  |  |  |
|  | BRS-DLV score | 0.467 | 0.273 | 0.284 | 0.638 | 0.087 | 0.001 |  |  | 1.022 |
|  | Age | -0.003 | -0.028 | -0.013 | 0.007 | 0.005 | 0.593 |  |  | 1.081 |
|  | MHRR | 0.026 | 0.012 | -0.193 | 0.238 | 0.108 | 0.821 |  |  | 1.056 |
|  | Gender | -0.040 | -0.017 | -0.242 | 0.182 | 0.109 | 0.714 |  |  | 1.020 |
|  | BMI | 0.011 | 0.036 | -0.018 | 0.038 | 0.013 | 0.443 |  |  | 1.064 |
| 2 |  |  |  |  |  |  |  | 7.4% | 6.6% |  |
|  | Constant | 2.384 |  | 1.407 | 3.324 | 0.511 | 0.001 |  |  |  |
|  | BRS-DLV score | 0.469 | 0.274 | 0.299 | 0.634 | 0.088 | 0.001 |  |  | 1.018 |
|  | Age | -0.003 | -0.030 | -0.015 | 0.007 | 0.005 | 0.583 |  |  | 1.040 |
|  | Gender | -0.040 | -0.017 | -0.256 | 0.176 | 0.109 | 0.711 |  |  | 1.019 |
|  | BMI | 0.010 | 0.035 | -0.016 | 0.040 | 0.014 | 0.446 |  |  | 1.060 |
| 3 |  |  |  |  |  |  |  | 7.4% | 6.8% |  |
|  | Constant | 2.376 |  | 1.318 | 3.358 | 0.527 | 0.001 |  |  |  |
|  | BRS-DLV score | 0.466 | 0.272 | 0.290 | 0.644 | 0.088 | 0.001 |  |  | 1.008 |
|  | BMI | 0.010 | .0.34 | 0.570 | 0.039 | 0.015 | 0.507 |  |  | 1.048 |
|  | Age | -0.003 | -0.030 | 0.534 | 0.008 | 0.005 | 0.534 |  |  | 1.039 |
| 4 |  |  |  |  |  |  |  | 7.3% | 6.9% |  |
|  | Constant | 2.293 |  | 1.285 | 3.349 | 0.499 | 0.001 |  |  |  |
|  | BRS-DLV score | 0.465 | 0.272 | 0.288 | 0.288 | 0.084 | 0.001 |  |  | 1.008 |
|  | BMI | 0.008 | 0.028 | -0.014 | -0.014 | 0.013 | 0.547 |  |  | 1.008 |
| 5 |  |  |  |  |  |  |  | 7.3% | 7.0% |  |
|  | Constant | 2.523 |  | 1.843 | 3.219 | 0.336 | 0.001 |  |  |  |
|  | BRS-DLV score | 0.461 | 0.269 | 0.293 | 0.626 | 0.089 | 0.001 |  |  | 1.000 |

*BRS-DLV = Brief Resilience Scale-Dutch Language Version. UWES =Utrecht Work Engagement Scale; the construct vitality of this scale was used. BMI = Body Mass Index. MHRR = Mean Heart Rate Range, the measure for Heart Rate Variability.*
